# Is biological repair of heart on the horizon?

**DOI:** 10.12669/pjms.334.12938

**Published:** 2017

**Authors:** HR Ahmad, Satwat Hashmi

**Affiliations:** 1 Dr. HR Ahmad, MD PhD Bochum, FCPS. Department of Biological and Biomedical Sciences, Aga Khan University, Karachi, Pakistan; 2 Dr. Satwat Hashmi, MBBS MS PhD. Department of Biological and Biomedical Sciences, Aga Khan University, Karachi, Pakistan

**Keywords:** Stem cells, Cardiomyocytes and Cardiac regeneration

## Abstract

The stem cells keep us young by endogenously repairing us upon need. They do so bysmartly one step forward towards differentiation while another step backward to nurturethe undifferentiated stem cells. They are building blocks for every organ witha differential rate of repair of worn out tissues. Since stem cells can be cultured with a normal karyo type, they could be the ideal source for heart repair after myocardial infarction. As opposed to lower vertebrates and neonatal mice, cardiac regeneration in adult mammalian heart seems to be difficult to assess with a solid evidence of cytokinesis. It becomes more difficult to quantify the level of regeneration after myocardial infarction injury against a background of a large invasion of proliferating inflammatory cells. The question to beraised is how the renewal of a piece of myocardium follows the time line of picking upcell types in series: cardiomyocytes, endothelial cells, smooth muscle cells, fibroblast, pacemaker cells, conducting and Purkinje cells to bring the orchestration of rhythmically contracting and relaxing heart. This review focuses on where we are onthe status of heart repair and cardiac regeneration.

## INTRODUCTION

Human embryonic stem cells within blastocyst have made us what we are. The stem cells keep us young by endogenously repairing us upon need. They do so by smartly one step forward towards differentiation while another step backward to nurture the undifferentiated stem cells. This clinic is on-line and free for all target organs. Thus, they are building blocks for every organ with differential rate of repair of worn out tissues. It was the pioneering work of Wilmut et al.^1^ and Thomson et al.^2^ on the *cloning* and the identification of human *embryonic stem cells* that initiated a new era for research in molecular stem cell biology leading to therapeutic potential. This review focuses on where we are on the status of heart repair and cardiac regeneration.^3^

### What is the scope of cardiacstem cell research?

**Fig. 1:**
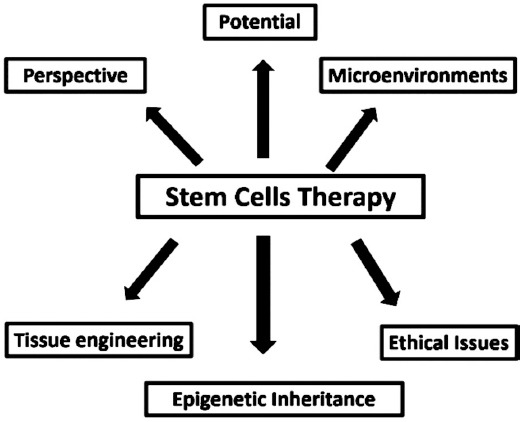
Scope of stem cell research.

Fig. 1 shows the components of this scope:

1) ***Perspective:*** It is aimed to provide cell replacement therapy to halt progressive heart failure and repair heart after myocardial infarction (MI).

### Potential

Since stem cells can becultured with a normal karyotype and regenerative potential, they could be the source for heart repair after MI.

### Microenvironment

Since stem cells are controlled by a particular microenvironment, it is supported by feeding cells, basement membrane and biologically active molecules. Understanding the stem cell-environment-interaction at the cellular, molecular and genetic levels forms the basis of biological repair of heart. The discovery of neural/cardiac stem cells has opened new opportunity of biological repair of brain and heart. However, they seem to be restricted regionally and temporally. It means that not all stem cells are equivalent. How good the bone marrow stem cells could home to heart to become the part of the working myocardium has been tried but with limited success. How long they would remain true to myocardium is not warranted.

2) ***Tissue Engineering:*** careful tissue engineering is required to produce an ideal mix of stem cells for the biological repair of diseased organs. As stem cells have the tendency to be transformed into cancer cells, it could be considered as one of the sources of tumor-genesis in different target organs.^4^

3) ***Epigenetic inheritance:*** Of note is the fact that the genotypes of most cells aresimilar. Yet they show diversity in morphology [phenotype] and functions. It is because the selective expression and repression of genes that determine the specific properties of individual cells. What are signals that can reprogram differentiated cell back to totipotency need to be fully elucidated?

4) ***Ethical issues:*** Since resources of acquiring stem cells are many, ethical and social consideration of biological repair might pose with difficulty. Therefore, any use of stem cell therapy should be subjected to appropriate scientific and ethical review committee.

### Is human heart post-mitotically locked?

**Fig. 2:**
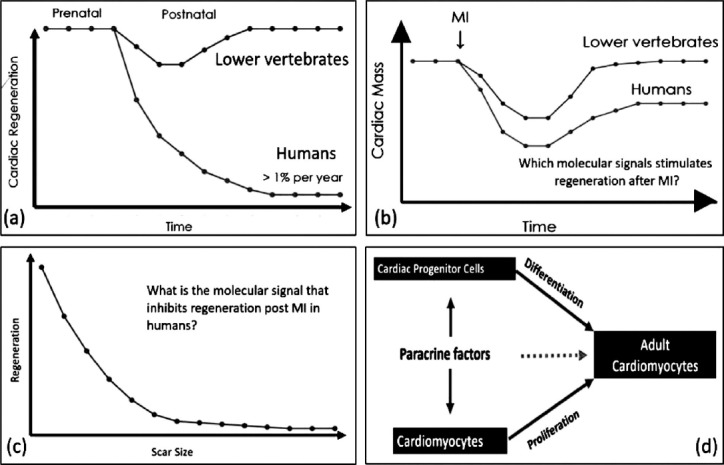
Schematic representation of key concepts in biological repair of heart.

We thought the mammalian heart to be post mitotically locked to live as long we live.^5^ However, this has been challenged with evidence ofresident/homing stem cells for cardiac regeneration. Prenatally, this phenomenon is working well. However, this mechanism is switched off postnatal showing the stage of terminal differentiation. This transition might be associated with the transformation of hyperplasia to hypertrophy. DNA replication study with evidence of cytokinesis in cardiomyocytesis actively sought as a test of proliferation and regeneration.^6^,^7^ Since cytokinesis could not be evidenced in most studies, aquantitation of regeneration is difficult to assess.^8^,^9^ Loss of cardiac mass as a result of MI or progressive heart failure is a major cause of morbidity and mortality.^10^ Since human heart is persistently scarred after MI, the endogenous cardiac regeneration seems to be inhibited by the scarformation^11^,^12^ (Fig. 2c).

### Can heart be regenerated by cardiac resident and homingstem cells?

Findings in lower vertebrates and neonatal mice are encouraging but cardiac regeneration in adult mammalian heart seems tobe harder to assess with solid evidence (Fig. 2a and b). It becomes more difficult to quantify the level of regeneration after MI injury with a background of a large invasion of proliferating inflammatory cells. What are tools to assess cardiac regeneration capacity? These include DNA synthesis labeling, transgenic mouse modelsand genetic lineage tracing based on molecular markers, multi-isotope imaging mass spectrometryand immunohistochemistry and flow cytometry. Addition of nucleosides into the newly formed DNA shows a rate of less than 1% of cardiac regeneration per year. Non-cardiomyocyte cellslike fibroblasts, endothelial cells, smooth muscle cell and immune cellsand lack of evidence of cytokinesis due to rigid sarcomeric membrane seem to be confounder how good the regeneration rate could be estimated.^13^ Interestingly, various methods using a transgenic mouse model with beta-galactosidase, 14C labelingof cardiomyocyte nucleiand multi-isotope imaging mass spectrometryshowed a renewal rate not more than 1-2% per year.^6^,^8^,^9^ However, to what extent these rates might increase after injury is not clear. Comparing mice with human’s rate of renewal in context of life span, this low level regenerative capacity may be of significance in humans for on line acute repair of heart.

The question to be raised is how the renewal of a piece of myocardium follows the time line of picking up cell types in series: cardiomyocytes, endothelial cells, smooth muscle cells, fibroblast, pacemaker cells, conducting and Purkinje cells to bring the orchestration of rhythmically contracting and relaxing heart. How do genes encode these various cellular programs need to be decoded bywell-designed genomic and proteomic methodologies. This experimentation might show if cardiomyocyte genome contained a dormant stem cell regeneration site that could be coaxed for the biological repair of heart.^14^,^15^

### What are cellular sources of adult cardiac regeneration?

Since the DNA-labeling studies did not show the cellular source of regeneration in the adult or injured heart, cell transplantation and genetic lineage tracing based on molecular markers were tried. Orlic et al.^16^ were the first to show a 68% regeneration of the infarcted myocardium in rodents in response to injection of Lin-c-Kit+ BMCs [Bone marrow cells] with a delay of 9 days. This showed that it took 9 days for processing the BMCs to cardiac cellular commitment, differentiation and functional maturation. The sad story is that the claims of this study could not be reproduced no matter how the cells were isolated and treated.^17^ BMCs could have provided paracrine cardioprotective factors to improve the cardiac function.^18^ What might be the underlying mechanisms are not known. Since cKit BMCs could not regenerate heart, cultured Lin-cKit+ cardiac progenitor cells, however, showed initially a positive response to regenerate 70% of adult rodent hearts after MI injury.^11^ This study was also not confirmed in adult hearts.^19^ Uchida et al^20^ reported Sca1-derived cells as a source of myocardial renewal in the murine adult heart. Further studies showed that Sca-1-expressing cells in the heart could be endothelial progenitors.^21^ How good could the Sca-1 lineage –traced CPCs contribute new cardiomyocytes to injured heart remains uncertain. However, the emerging consensus is that both c-Kit+ and Sca-1+ resident CPCs cannot differentiate and regenerate the heart with anew contracting myocardium. How to divert their endothelial programs into cardiomyocyte lineage remains to be seen. Another member walks into this family is cardiac – resident SPcellswith a molecular marker of ABC transporter proteins being able to transdifferentiate into cardiomyocytes, endothelial cells and smooth muscle cells.^22^ These three marker proteins can be used to identify specific population of CPCs to be coaxed into the cardiomyocyte lineage. Of noteis the fact that during embryonic development the true cardiac progenitors Is1+ cells can give riseto all lineages found in the heart.^23^ Similarly epicardially derived cells differentiate into cardiomyocyte. How these prenatal programs could be activated in the adult heart as to be capable of generating new cardiomyocytes, are not elucidated.^24^ Genetic lineage tracing with 15N-thymidine labeling followed by imaging mass spectroscopy showed that the mammalian heart can be regenerated from preexisting cardiomyocytes however, being limited in potential.^9^ These cardiomyocytes might be involved in ongoing renewal, but their response to the extent of MI seems to be blunted. Since these cardiomyocytes are also refractory to cytokinesis, response to MI might depend on this. Factors blocking cytokinesis could be a genetic block, overexpression of cyclin-D2, Rb proteins deficiency switching off cardiomyocyte cell cycle activity and the quantity ofnucleation.^13^,^25^ These factors regulating molecular switches ofcardiomyocyte proliferation with a normal cytokinesis should be experimentally understood as to how scar formation prevents regeneration of myocardium in response to MI.

### What are challenges in biological repair of heart?

The real challenge is to gain insights of dynamics of fetal to adultcardiomyocyte gene expression encoding proteomics for a well-organized sarcomeres, t-tubules, and ion channels to ensure an endogenous repair of adult heart. The delivery method of cardiac repair is causing uncertainties in context of either an intravenous injection or direct coronary infusion of stem cells. The cellular engraftment rate of injected cells is rather low. Paracrine mediators likeextra cellular matrix factorsangiogenic growth factorsand interleukins among others could stimulate regeneration from resident cardiac stem cells and/orcardiomyocytes. How rapidly newly formed cardiomyocytes mature in vivo/in vitro could provide restoration of cardiac function with less risk of arrhythmias? How to use and interpret the time line of cardiomyocyte progeny with essential cell lines of a beating myocardium? How to achieve this mission against the tide of risk factors of rejection, cancer conversion and maturity are challenges to understand and develop means of rectification for good news from the clinical trials.

### What are implications for biological repair of heart?

Since the acute biological repair of adult heart seems to be driven mainly by the proliferation of cardiomyocytes and to a lesser extent by transdifferentiation of cardiac progenitor cells [CPC], future line of action should be to therapeutically focus on these two pathways (Fig. 2d). Alternative cell replacement therapies [CRT] should be sought to enhance the regenerative capacity of the myocardium with all functional cell lines enabling to join the orchestra of a beating heart. Trials with overall survival as an endpoint are awaited to ensure CRT to be adopted for the cost effective biological repair of heart.

### What can we learn from cardiac progenitor cell-based trials?

There is an interesting race between preclinical models and clinical trials. The award of the former depends on the later. Bolliet al.^26^ reported the first trial on autologous cKit+ CPCs from cardiac tissue being harvested during CABG. These cells were cultured and then injected through arterial bypass graft. Large size trial has been recommended with a cocktail of c-Kit+ CPCs and bone marrow derived stem cells to reduce infarct size and restore cardiac function after MI.^27^ Since the engraftment of transplanted cells remains low in most of the clinical trials so far, the potential benefits could be due to paracrine effect as opposed to transdifferentiation of injected cells into cardiomyocytes.^28^ Might it be possible to study the constituents of the paracrine mediators to repair heart instead of cell replacement therapy? On the front line are ES (embryonic stem) and iPS(induced pluripotent stem) cells to compete for biological repair of heart. Pre-differentiated-ES-patch has been aimed to align cardiomyocytes with the ECM to ensue contractile force after adherence to the native porcine myocardium.^29^ Human ESC-derived-cardiomyocyteshave been shown to regenerate non-human primate hearts.^30^ The question arises is might it be possible to switch fibroblast in the MI-scar into the cardiomyocytes? Direct reprogramming of fibroblast into functional cardiomyocytesby factors such as micro-RNAhas been shown by Iedaet al.^31^ andJayawardena et al.^32^ How far these results frompreclinical animal models could be transferred to human are challenges of heart repair depending upon good news from the clinical trials with the endpoint of appreciable survival

(a) Shows time course of pre and post natal cardiac regeneration between lower vertebrates and human. Prenatally both species are regenerative friendly. Humans dissociate markedly from lower vertebratespostnatally.
(b)Is a cardiac mass response to MI between two species showing complete recovery in lower vertebrates.
(c) Shows hyperbolic relationship between regeneration and scar size in response to MI.
(d) Is a model showing how adult cardiomyocytes might regenerate in response to injury?
